# Neurofilament Light Chain: A Candidate Biomarker of Perioperative Stroke

**DOI:** 10.3389/fnagi.2022.921809

**Published:** 2022-07-07

**Authors:** Xiaoting Zhang, Huixian Wang, Li Li, Xiaoming Deng, Lulong Bo

**Affiliations:** ^1^Faculty of Anesthesiology, Changhai Hospital, Naval Medical University, Shanghai, China; ^2^Department of Anesthesiology, Affiliated Beijing Friendship Hospital, Capital Medical University, Beijing, China

**Keywords:** perioperative, stroke, neurofilament light chain, expression, biomarker

## Abstract

Perioperative stroke is defined as a brain infarction of ischemic or hemorrhagic etiology that occurs during surgery or within 30 days after surgery. However, identifying perioperative stroke is challenging. Thus, the discovery and validation of neurological biomarkers for perioperative stroke are urgently needed. Neurofilament forms part of the neuronal cytoskeleton and is exclusively expressed in neurons. After disease-related neuroaxonal damage occurs, neurofilament light chain protein is released into the cerebrospinal fluid and blood. Blood neurofilament light chain has recently been shown to serve as a potential marker of interest during the perioperative period. Therefore, the aim of the present review was to give an overview of the current understanding and knowledge of neurofilament light chain as a potential biomarker of perioperative stroke.

## Introduction

With the development of an aging population, surgical case volumes have gradually increased annually. Compared with other perioperative complications, perioperative stroke is a potentially devastating, and relatively under-recognized neurological complication of surgery with high mortality and morbidity. The incidence of perioperative stroke varies among different types of surgical procedures and populations. It is approximately 0.1–1.9% in patients undergoing non-cardiac or non-neurological surgery, compared to 1.9–9.7% in patients undergoing high-risk cardiovascular or neurological surgery (Al-Hader et al., 2019). Compared to community-onset stroke, perioperative stroke is a serious medical emergency, represents a significant public health burden, and usually has unfavorable outcomes (Benesch et al., 2021).

Based on the guidelines of the Society for Neuroscience in Anesthesiology and Critical Care, perioperative stroke is defined as a brain infarction of ischemic or hemorrhagic etiology that occurs during surgery or within 30 days after surgery (Vlisides et al., 2020). Perioperative stroke can develop intraoperatively or postoperatively after recovery from anesthesia (Vlisides et al., 2020). However, identifying perioperative stroke is sometimes challenging due to its pathogenesis is multifactorial. The evaluation of neurological outcome of patients can sometimes be affected by the residual effects of anesthetic agents and medications, certain underlying metabolic causes, and symptoms related to cerebral irritation such as delirium and agitation (Misal et al., 2016).

Many risk factors have been identified and associated with perioperative stroke such as age, previous stroke, male sex, obesity, diabetes mellitus, atrial fibrillation, hypertension, smoking, physical inactivity, and acute stress (Nagre, 2018). Several screening tools for stroke have been available for the postoperative period such as the modified National Institutes of Health Stroke Scale (mNIHSS), although its validity has not been verified in surgical populations (Sun et al., 2016). Thus, the prevention, early identification, and proper management of perioperative stroke are of vital importance.

A biomarker is a defined characteristic that is measured as an indicator of normal, biological, pathogenic processes, or responses to an exposure or intervention (Califf, 2018). Considerable interest exists in reliable neurological biomarkers with the hope that they will improve the accuracy of differential diagnosis and prognostic assessment, as well as predict the severity of the adverse outcomes of perioperative stroke. Thus, the discovery and validation of neurological biomarkers for perioperative stroke are urgently needed, which will be useful for subsequent clinical management.

Neurofilament forms the neuronal cytoskeleton and is exclusively expressed in neurons. After disease-related neuroaxonal damage occurs, neurofilament light chain (NfL) protein is released into the cerebrospinal fluid (CSF) and, to a smaller extent, in the peripheral blood (Khalil et al., 2018; Uphaus et al., 2019). Accumulating evidence has illustrated that NfL changes more significantly in the occurrence and development of various nervous system diseases, which indicates that NfL is an emerging marker of neuronal and axonal injury, and thus has important significance for the diagnosis and prognosis of various diseases (Teunissen et al., 2022). Blood levels of NfL has recently been shown to serve as a potentially interesting marker during the perioperative period. Therefore, the aim of the present review was to give an overview on the current knowledge and understanding of NfL as a potential biomarker of perioperative stroke.

## Structure, Function, and Measurement of Neurofilament Light Chain

Neurofilament heteropolymers are major cytoskeletal components of axons. They are responsible for axoplasmic transport, thereby maintaining the normal morphology of neurons and the elasticity of nerve fibers, preventing their breakage, and promoting the radial growth of axons (Lee et al., 1993). A neurofilament is composed of an NfL, a neurofilament medium chain (NfM), and a neurofilament heavy chain (NfH). The C-terminal of the NfM and NfH has 483 amino acids and 676 amino acids, respectively; therefore, the molecular weight of NfL is far less than that of NfM and NfH, which is 68, 150, and 190–210 kDa, respectively (Yuan et al., 2012).

NfL is the only neurofilament protein that can self-assemble functional fibers. Moreover, the abnormal expression of NfL and the mutation of human NfL gene can cause a variety of diseases. A large number of clinical and experimental studies (Barro et al., 2020; Biernacki et al., 2022) have illustrated that the level of NfL changes more significantly in the occurrence and development of various nervous system diseases. This finding suggests that NfL is a molecular marker of neuronal and axonal injury, which has important guiding significance for the diagnosis and prognosis of a variety of neurological disorders.

NfL has emerged as a biomarker candidate for neurodegenerative pathology in a number of neurological diseases. Under pathological conditions, the amount of NfL released depends on the severity of axon injury and is released into peripheral blood at a low concentration. With the development and progress of immunoassay technology, remarkable progress has been made in the detection of NfL (Thebault et al., 2021). Accurate measurement of its concentration can help in the understanding of the development of many diseases. CSF sampling is clinically a traumatic examination, and CSF samples are not easy to obtain as are peripheral blood samples. Previous detection methods have primarily used enzyme-linked immunosorbent assay to determine the concentration of NfL in CSF. Since the concentration of NfL in blood is only 1/40 of the content of CSF, the sensitivity of the ELISA assay is not enough and it is difficult to meet the requirements when used in the detection of blood NfL. With the introduction of third-generation electrochemiluminescence technology, the analytical sensitivity has been greatly improved (Zanut et al., 2020). In recent years, the latest generation of single-molecule array (SiMoA) technology allows the recognition resolution of ultra-high molecular signal and the detection of single molecule through digital signal output form. It is convenient for the wide application of blood NfL detection for clinical and experimental use. With the advent of SiMoA technology, reliable quantification of low NfL concentrations in the peripheral blood became available (Kuhle et al., 2016). This cutting-edge technique facilitated the comprehensive study of this protein as a diagnostic and prognostic marker in various acute and chronic neurological disorders.

Factors other than neuroaxonal damage may also contribute to the increase in blood NfL is important to highlight. NfL increases in normal aging, paralleled by a higher variability in the elderly population. Thus, correctly interpreting this marker on an individual level is difficult. Some evidence also suggests that blood NfL may be influenced by body mass index, blood volume, and renal function. Koini et al. (2021) explored factors influencing the blood NfL concentration by analyzing a large set of demographic, lifestyle, and clinical variables in a normal aging cohort that included 327 neurologically inconspicuous individuals. Their results showed that age was, by far, the most important factor influencing blood NfL concentration, which was primarily driven by individuals ≥ 60 years. In individuals ≥ 60 years, age, blood volume, renal function, and high density lipoprotein were associated with blood NfL levels, although at a much lower scale to influence blood NfL. Body mass index independently predicted blood NfL in individuals aged 38–60 years (Koini et al., 2021). A speculation is that the increase in blood NfL in a normal aging cohort was associated with brain volume loss over time, thereby supporting the notion that other factors beyond normal aging may also contribute to the increase in blood NfL concentrations in older individuals (i.e., > 60 years; Khalil et al., 2020).

NfL is a sensitive but non-specific marker of brain injury. Fohner et al. explored the association of blood NfL and magnetic resonance imaging (MRI) findings in older adults. Their longitudinal cohort study included two cranial MRI scans conducted approximately 5 years apart and assessed for white matter hyperintensities and infarcts. Among older adults without a history of stroke, higher blood NfL concentration was associated with the covert MRI findings of vascular brain injury, especially the burden of white matter hyperintensities and its worsening (Fohner et al., 2022).

## Neurofilament Light Chain and Perioperative Stroke

For surgical patients, especially those at high risk of stroke, the early detection of perioperative stroke will greatly benefit the patient’s perioperative prognosis and functional outcomes (Ko, 2018). Patients with symptoms of transient ischemic attacks and clinical signs of stroke constitute only a small fraction of patients experiencing perioperative stroke. Perioperative stroke can occur in the forms of overt or covert stroke, with the latter being dominant; therefore, many patients with perioperative stroke are not identified or detected in time. Diffusion-weighted MRI has high sensitivity and specificity in relevant changes in brain imaging. However, this method is time-consuming, expensive, and of limited value for repeated monitoring, which may limit its clinical use. Several neurological biomarkers have been identified and their use during the perioperative period has been explored. More in-depth studies of neurological biomarkers may help to identify patients at high risk of perioperative stroke, including neurological impairment and vulnerability.

Learning from previous studies on biomarkers of community-onset stroke is worthwhile and can provide hints for identifying biomarkers for perioperative stroke. A series of studies (Pedersen et al., 2019; Pekny et al., 2021) indicate that blood NfL levels are increased in the acute phase after stroke and peak during the initial 3 months. A few studies (Stokowska et al., 2021) have demonstrated that blood NfL can serve as a predictor of functional improvement in the late phase after stroke. Patients with higher blood NfL, compared to patients with lower blood NfL, had a 1.71 times higher risk of poor functional outcomes during follow-up after ischemic stroke (Liu et al., 2020). A recent review summarized the findings of recent literature exploring the blood NfL level in the acute and post-acute phase after stroke (Pekny et al., 2021). Blood NfL also serves as a predictor of adaptive neural plasticity and functional improvement in the late phase after stroke. A conclusion is that high blood NfL levels in the acute phase after stroke can predict unfavorable outcomes. However, previous studies have rarely evaluated the role of NfL in perioperative periods, especially its role in perioperative stroke.

Chen et al. (2021) found that blood NfL predicts the outcomes in stroke patients receiving endovascular thrombectomy (EVT). Their study included 60 patients receiving EVT for acute ischemic stroke with large vessel occlusion type and focused on changes in the levels of blood NfL, GFAP, tau, and ubiquitin carboxyl-terminal esterase L1 before EVT, immediately after EVT, and 24 h after EVT. Higher blood NfL levels before, immediately after EVT, and 24 h after EVT were associated death or disability at 90 days.

Rahmig et al. (2021) in their prospective study analyzed blood NfL levels in patients undergoing EVT for anterior circulation large vessel occlusion. The blood NfL level was serially measured before EVT and at 30 min, 6 h, 12 h, 24 h, 48 h, and 7 days after EVT. No differences existed among the serial blood NfL levels in the first 12 h post-EVT; however, a constant increase was observed afterward. Serum NfL may complement clinical and imaging predictors of treatment response and functional outcome in stroke patients undergoing EVT for anterior circulation large vessel occlusion (Rahmig et al., 2021).

Alifier et al. (2020) found that patients who had cardiac surgery had higher blood NfL levels than did patients who underwent other operations and that patients who experienced cardiopulmonary bypass had even higher levels. Their results indicated that blood NfL levels may guide the development of surgical procedures to minimize neuronal damage and may be used in longitudinal clinical studies to evaluate the relationship of surgery with future neurocognitive impairment. Saller et al. (2019) observed a sharp postoperative increase in blood NfL in cardiac surgery patients with cardiopulmonary bypass with the highest levels occurring in patients with delirium. A speculation is that surgery or trauma can induce a neuroinflammatory response and microglia activation, thereby eventually leading to neuronal damage. Thus, blood NfL may be of benefit to identify cardiac surgery patients at risk of delirium and to detect individuals with the postoperative emergence of delirium.

A recent study (Zhang et al., 2021) indicated that the median blood NfL level of patients with an acute type A aortic dissection who had a stroke 12 h after surgery was nearly double that of patients who did not have a stroke postoperatively. Patients with stroke had a higher baseline value than did non-stroke patients. Their study interestingly did not find changes in S100β protein or in neuron-specific enolase in patients with stroke. To the best of our knowledge, the Zhang study was the first to evaluate the role of blood NfL as an early and sensitive biomarker for predicting perioperative stroke, particularly 12 h after surgery. Thus, further studies are needed to validate whether the blood NfL can be used as a biomarker for postoperative stroke.

Whether blood NfL is better than other biomarkers such as S100 and neuron-specific enolase in the prediction and prognosis of perioperative stroke would also be interesting to know. No study has perioperatively compared blood NfL and other biomarkers, although the findings from studies on community-onset stroke could provide valuable insights. The area under the curve of blood NfL for the prediction of cardiovascular events and functional outcome 90 days after acute ischemic stroke has been reported as significantly higher than that of S100, N-terminal pro-brain natriuretic peptide, atrial natriuretic peptide, and fatty acid–binding proteins (Uphaus et al., 2019). A recent study (Chen et al., 2021) reported that blood NfL, tau, and glial fibrillary acidic protein increased over time in patients after EVT in anterior circulation stroke. Blood tau and glial fibrillary acidic protein levels peaked at 24–72 h and were lower at 3 months after stroke, whereas blood NfL levels continued to increase at 3 months, which makes it a promising biomarker for prognostic prediction after stroke.

Covert strokes represent brain infarcts that are not recognized acutely because of unappreciated, subtle, or misclassified manifestations but are detected on brain imaging. Covert stroke is more common than overt stroke in the non-operative setting, which raises the possibility that covert strokes may occur after non-cardiac surgery. The NeuroVISION study (Mrkobrada et al., 2019) was a prospective cohort study conducted in 12 academic centers in nine countries. In the study, 1114 patients aged 65 years or older who underwent in-hospital, elective, non-cardiac surgery were included and underwent brain MRI after surgery. The NeuroVISION study demonstrated that 7% of patients had a perioperative covert stroke, which was associated with an increased risk of perioperative delirium and cognitive decline at 1 year (Mrkobrada et al., 2019). However, the availability of MRI scanners may limit the immediate evaluation of underlying perioperative stroke. Casey et al. found that postoperative delirium, occurred at least once during the postoperative periods of 1–4 days, is associated with a greater rise in NfL on postoperative day 1 than in participants who did not have delirium (Casey et al., 2020).

The Casey study suggests that the incidence of perioperative covert stroke is relatively high and may have been underestimated previously. It also identified a link between perioperative covert stroke and cognitive decline in surgical populations. Thus, research aimed at determining whether postoperative delirium is the manifestation form of perioperative covert stroke is attracting increasing research interest. If appropriate biomarkers are available, patients at risk can be timely screened and accurately assessed preoperatively, thereby allowing for a more accurate and desirable anesthesia plan.

Recent data exploring the potential role of covert perioperative stroke in postoperative delirium and cognitive function have opened up a whole new range of research area (Cui et al., 2020). Evaluating the neurological condition of postoperative patients is usually difficult because of sedation or mechanical ventilation and because moving these patients may be inappropriate. Thus, a promising and potential biomarker reflecting brain damage will be of great value and provide valuable information to help in the clinical management of patients.

The Successful Aging after Elective Surgery Study examined the association of the levels of plasma NfL, total tau, glial fibrillary acid protein, and ubiquitin carboxyl-terminal hydrolase L1 with delirium, delirium severity, and cognitive performance in older adults without dementia who are undergoing major elective surgery (Fong et al., 2020). The results indicated that patients with delirium had higher NfL levels postoperatively. Patients with the highest preoperative or postoperative day 2 blood NfL levels were more likely to develop into delirium. Elevated blood NfL at 1 month after surgery was associated with delirium and greater cognitive decline. These findings suggest that blood NfL may be useful as a biomarker for predicting the risk of delirium and long-term cognitive decline and, once confirmed, would provide pathophysiological insights and new evidence on neuroaxonal injury after delirium (Fong et al., 2020).

Results from the Cerebrospinal Fluid and Preclinical Alzheimer Cognitive Trajectory (CAPACITY) and the Assessment and Review of Cognition, Alzheimer’s Disease, and Inflammation in Elderly Patients After Hospital Intervention (ARCADIAN) studies found that the mean blood NfL increased postoperatively to a maximal level at 48 h (Evered et al., 2018). Tau levels also increased significantly from baseline with a peak increase at 6 h postoperatively, after which they declined but remained elevated for at least 48 h. A significant and rapid increase in blood NfL and tau occurred in response to anesthesia and surgery, regardless of the type of anesthesia or surgery. The changes in blood NfL reflect an acute response to the precipitating event of anesthesia and surgery, which may be more similar to acute traumatic brain injury. These findings indicate that general anesthesia and surgical procedures exert acute response to neuronal injury on the central nervous system, which has enlightenment for neurological outcomes and for the mechanisms underlying general anesthesia (Evered et al., 2018).

## Conclusion and Future Directions

Recognition of perioperative stroke is often difficult, particularly in the early stages. Perioperative stroke can present with only impaired consciousness and is usually in the differential diagnosis of delayed emergence after general anesthesia. A large knowledge gap remains with regard to perioperative stroke. The identification of reliable and effective biomarkers for perioperative stroke is an urgent and challenging task in clinical practice. Clinicians are more inclined to choose more effective and affordable screening methods for perioperative stroke, other than MRI scans.

An ideal biomarker should be accurate, non-invasive, and inexpensive. It should be easily measured in a standardized manner with a sensitivity and specificity of at least 80%. An important factor is that clinical management strategies could be determined, based on biomarker levels. Studies evaluating the role of perioperative NfL remain insufficient, although NfL levels measured during the perioperative period shows potential in identifying patients at risk of perioperative stroke, postoperative delirium, and cognitive dysfunction. Also worth considering is that the blood NfL level, as a biomarker of neuronal injury, which universally occurs after damage to nervous system, will increase in various neurological diseases. Nevertheless, blood NfL cannot be used to differentiate between diseases without the aid of clinical examinations and other laboratory and imaging studies. Furthermore, to understand the dynamics of blood NfL perioperatively, as well as its maximum serum concentration and half-life in blood, is of great significance. No clear answer to these questions exist. This issue needs to be clarified by further research.

To conclude, the measurement of blood NfL levels in clinical practice provides a relatively simple and quantitative method to measure neuronal damage. Future studies will better define its optimal use in clinical practice, including the establishment of its reference range and a more complete understanding of the regulating mechanism responsible for the release of NfL in perioperative stroke.

## Author Contributions

XZ, HW, and LB contributed to the search and assessment of the available literature, and wrote the manuscript. LL and XD helped to revise the manuscript to the final form. All authors contributed to the article and approved the submitted version.

## Conflict of Interest

The authors declare that the research was conducted in the absence of any commercial or financial relationships that could be construed as a potential conflict of interest.

## Publisher’s Note

All claims expressed in this article are solely those of the authors and do not necessarily represent those of their affiliated organizations, or those of the publisher, the editors and the reviewers. Any product that may be evaluated in this article, or claim that may be made by its manufacturer, is not guaranteed or endorsed by the publisher.

## Abbreviations

CSF: cerebrospinal fluid
EVT: endovascular thrombectomy
NfH: neurofilament heavy chain
NfL: neurofilament light chain
NfM: neurofilament medium chain.
